# Demonstrating the Impact of the Adsorbate Orientation on the Charge Transfer at Organic–Metal Interfaces

**DOI:** 10.1021/acs.jpcc.1c01306

**Published:** 2021-04-27

**Authors:** Thomas Georg Boné, Andreas Windischbacher, Marie S. Sättele, Katharina Greulich, Larissa Egger, Thomas Jauk, Florian Lackner, Holger F. Bettinger, Heiko Peisert, Thomas Chassé, Michael G. Ramsey, Martin Sterrer, Georg Koller, Peter Puschnig

**Affiliations:** † Institute of Physics, University of Graz, 8010 Graz, Austria; ‡ Institute of Physical and Theoretical Chemistry, University of Tübingen, 72076 Tübingen, Germany; § Institute of Organic Chemistry, University of Tübingen, 72076 Tübingen, Germany; ∥ Institute of Experimental Physics, Graz University of Technology, 8010 Graz, Austria

## Abstract

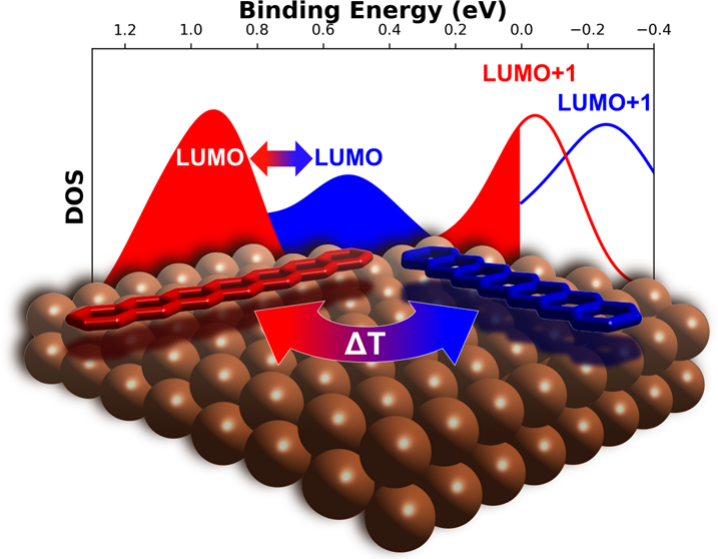

Charge-transfer processes at molecule–metal interfaces play a key role in tuning the charge injection properties in organic-based devices and thus, ultimately, the device performance. Here, the metal’s work function and the adsorbate’s electron affinity are the key factors that govern the electron transfer at the organic/metal interface. In our combined experimental and theoretical work, we demonstrate that the adsorbate’s orientation may also be decisive for the charge transfer. By thermal cycloreversion of diheptacene isomers, we manage to produce highly oriented monolayers of the rodlike, electron-acceptor molecule heptacene on a Cu(110) surface with molecules oriented either along or perpendicular to the close-packed metal rows. This is confirmed by scanning tunneling microscopy (STM) images as well as by angle-resolved ultraviolet photoemission spectroscopy (ARUPS). By utilizing photoemission tomography momentum maps, we show that the lowest unoccupied molecular orbital (LUMO) is fully occupied and also, the LUMO + 1 gets significantly filled when heptacene is oriented along the Cu rows. Conversely, for perpendicularly aligned heptacene, the molecular energy levels are shifted significantly toward the Fermi energy, preventing charge transfer to the LUMO + 1. These findings are fully confirmed by our density functional calculations and demonstrate the possibility to tune the charge transfer and level alignment at organic–metal interfaces through the adjustable molecular alignment.

## Introduction

When organic molecules adsorb on a metal surface, a number of processes such as molecular conformational changes,^1,2^ substrate reconstructions,^3,4^ or chemical reaction pathways^5^ are related to the realignment of the electronic levels at the interface. In general, these are accompanied by a redistribution of charges, for which several theoretical models have been developed depending on the strength of interaction between the adsorbate and the substrate.^6−10^ Regardless of the model, two factors can intuitively be identified when focusing on organic acceptors, i.e., molecules likely to uptake electrons. These are, on the one hand, the work function of the metal surface as a measure for the removal of electrons from the substrate and, on the other hand, the electron affinity of the molecule expressing its tendency to gain electrons. Given that the level alignment and thus charge transfer follow from intrinsic properties of the molecule and the substrate, only subtle changes induced by different adsorption configurations are to be expected for large organic adsorbates.^11−16^ Exceptions are often connected with severe phase changes where elongated planar molecules with their aromatic rings parallel to the surface (face-on) rotate to an upright standing configuration, with the aromatic system perpendicular to the substrate (edge-on).^17−21^

As many potential applications deal with the separation of charges, a thorough understanding of the interaction between the organic molecules and the metal contact is crucial for the design and tuning of applications.^4,22^ Thus, major research efforts have been directed toward the description of effects related to the electron transfer between a surface and its adlayer. Numerous experimental surface science techniques and computational approaches have been applied to investigate these interfaces. While well-known surface investigating techniques such as scanning tunneling microscopy (STM) and low-energy electron diffraction (LEED) primarily focus on determining structural aspects, (angle-resolved) ultraviolet photoemission spectroscopy (ARUPS) traditionally reveals the electronic (band) structure of the adsorbate layers. However, when utilizing the connection between ARUPS intensity maps and the Fourier transform of molecular orbitals, known as photoemission tomography (PT),^23^ ARUPS has been utilized to simultaneously reveal the electronic and geometric structure for a number of organic adsorbate systems.^16,24−29^

On the one hand, PT builds on the increasing availability of state-of-the-art electron energy analyzers, which are able to measure the photoemission intensity *I* over a wide range of binding energies *E*_B_ and parallel momenta components *k*_*x*_ and *k*_*y*_, respectively. On the other hand, it relies on the assumption that the final state of the photoemitted electron can be approximated by a plane wave, which works particularly well for flat aromatic systems where frontier orbitals are of π-character. Then, constant binding energy maps of the three-dimensional data cube *I*(*E*_B_, *k*_*x*_, *k*_*y*_) reveal the probability densities of Dyson orbitals of the initial state in momentum space that can often be approximated by molecular orbitals calculated in the framework of density functional theory (DFT).^30,31^ In particular, by analyzing momentum space signatures of the lowest unoccupied molecular orbital (LUMO) of the adsorbate, PT has been used to quantify the electron transfer from the substrate to the adsorbate system.^24,32−34^

With the intriguing optical and electronic properties of π-conjugated systems receiving increased attention in materials science,^35−40^ oligoacenes, i.e., aromatic hydrocarbons consisting of *n* linearly fused benzene rings,^41,42^ are a class of adsorbate molecules of renewed interest. The rod-shaped oligoacenes (*n*A) tend to form oriented monolayers on metal surfaces, and by varying the molecular length and/or the substrate, the electronic-level alignment can be tuned. While PT has already shed light on the behavior of tetracene (4A) and pentacene (5A) on coinage metals,^33,43^ recent advances have enabled access also to larger members of the family.^44^ For instance, we have succeeded in growing an ordered monolayer of the longer heptacene (7A) on Ag(110), despite its unfavorable reactivity toward oxidation and dimerization, thereby proving at the same time the orientated adsorption structure on Ag(110) and the charge transfer into the LUMO.^45^

In this work, we demonstrate that monolayers of heptacene on Cu(110) behave markedly different compared to all previously studied oligoacene monolayers on coinage metal surfaces, and, to the best of our knowledge, are also dissimilar to any other polycyclic aromatic hydrocarbons adsorbed on metal substrates studied so far.^16,26,46,47^ By using STM and combining PT with density functional theory (DFT) calculations, we show that not only the LUMO but also the LUMO + 1 receives charge when heptacene adsorbs face-on and orients along the Cu rows. Conversely, for heptacene still face-on but rotated by 90°, significantly less charge is transferred to the molecule, resulting in only the LUMO being filled and the molecular energy levels being shifted significantly toward the Fermi edge. These findings are fully confirmed by our DFT calculations and demonstrate that the charge transfer and level alignment at organic–metal interfaces not only depend on intrinsic properties of the adsorbate molecule and substrate but that the adsorption geometry, which could be tuned by suitable growth conditions, may play a crucial role.

## Experimental and Theoretical Methods

### Film Preparation and ARUPS Experiments

On Cu(110) crystals, thin layers of heptacene were created. Pristine Cu(110) single crystals were prepared by cycles of Ar^+^ sputtering (1 kV) and successive annealing (500 K, 5 min). The deposition of the heptacene molecule on the Cu(110) crystal was performed at three different temperatures. For cold sample preparations, the crystal was cooled to liquid nitrogen temperature (−198 °C). The hot sample preparations were performed at 250 °C. For the rest of the experiments, the sample temperature was equal to room temperature (25 °C). The deposition rates of the heptacene molecules were monitored with a quartz microbalance. Photoemission tomography measurements were performed using the NanoESCA system by ScientaOmicron. A helium gas excitation source was used at an energy of 21.22 eV (helium I line). For the calculation of work functions, the secondary electron cutoff and the Fermi edge were measured in a sample bias configuration. During photoemission tomography measurements, the sample temperature equaled room temperature.

### STM Experiments

The Cu(110) single crystal was cleaned by a standard procedure of two cycles of Ar^+^ sputtering and annealing. The sputtering was carried out at a voltage of 0.8 kV for 15 min at an argon partial pressure of 5 × 10^–5^ mbar, and the annealing was performed for 20 min at a temperature of 500 °C. The preparation of the crystal surface was confirmed by STM and LEED measurements. The molecules were evaporated on the crystal surface at rates of about 0.1–0.3 nm/min determined by a quartz microbalance. The STM and LEED measurements were performed in a two-chamber system with a variable-temperature (VT)-STM from Omicron NanoTechnology GmbH and a LEED/AES spectrometer from OCI Vacuum Microengineering Inc. at a base pressure of 3 × 10^–10^ mbar. Mechanically cut Pt/Ir tips were used for the STM measurements, and all tunneling voltages are given in relation to the sample. To improve the STM image contrast, we used the WSXM program, but no smoothing or altering was performed.^48^

### Density Functional Calculations

All calculations were performed within the framework of density functional theory (DFT) using the Vienna Ab Initio Simulation Package (VASP) version 5.4.4.^49,50^ Exchange–correlation effects were described by the functional of Perdew–Burke–Ernzerhof (PBE)^51^ and van der Waals contributions treated with the D3 dispersion correction.^52^ We utilized the projector-augmented wave (PAW) method^53^ together with an energy cutoff of 400 eV. The ionic positions of all structures were optimized within 10^–6^ eV with Gaussian smearing of 0.01 eV. For the monolayer of heptacene on Cu(110), the unit cell along the [11̅0] direction was derived from STM measurements. The model of heptacene in the [001] direction was constructed accordingly with the same intermolecular distances. The surface is simulated within the repeated slab approach using five metallic layers and a 30 Å vacuum layer. To avoid spurious electrical fields, a dipole layer is inserted in the vacuum region.^54^ The structure is optimized on a Monkhorst–Pack^55^ 5 × 2 × 1 grid of *k*-points constraining the coordinates of the two bottom Cu layers of the slab. The density of states of the total molecule–metal interface has been projected onto the molecular orbitals of the freestanding molecular layer in its distorted adsorption geometry, termed “molecular orbital projected DOS” (MOPDOS), following eq (1) of Lüftner et al.^56^ Subsequent to the geometry relaxation, the Kohn–Sham energies and orbitals are calculated non-self-consistently on a denser *k*-point mesh of 12 × 5 × 3, which is required for the simulation of the photoemission data. The angle-resolved photoemission intensity maps are calculated within the one-step model of photoemission^57^ approximating the final state as a plane wave,^43^ modified by an exponential damping factor introduced between the substrate and the organic molecule to mimic the mean free path of the photoemitted electrons.^56^

## Results and Discussion

**Figure 1:**
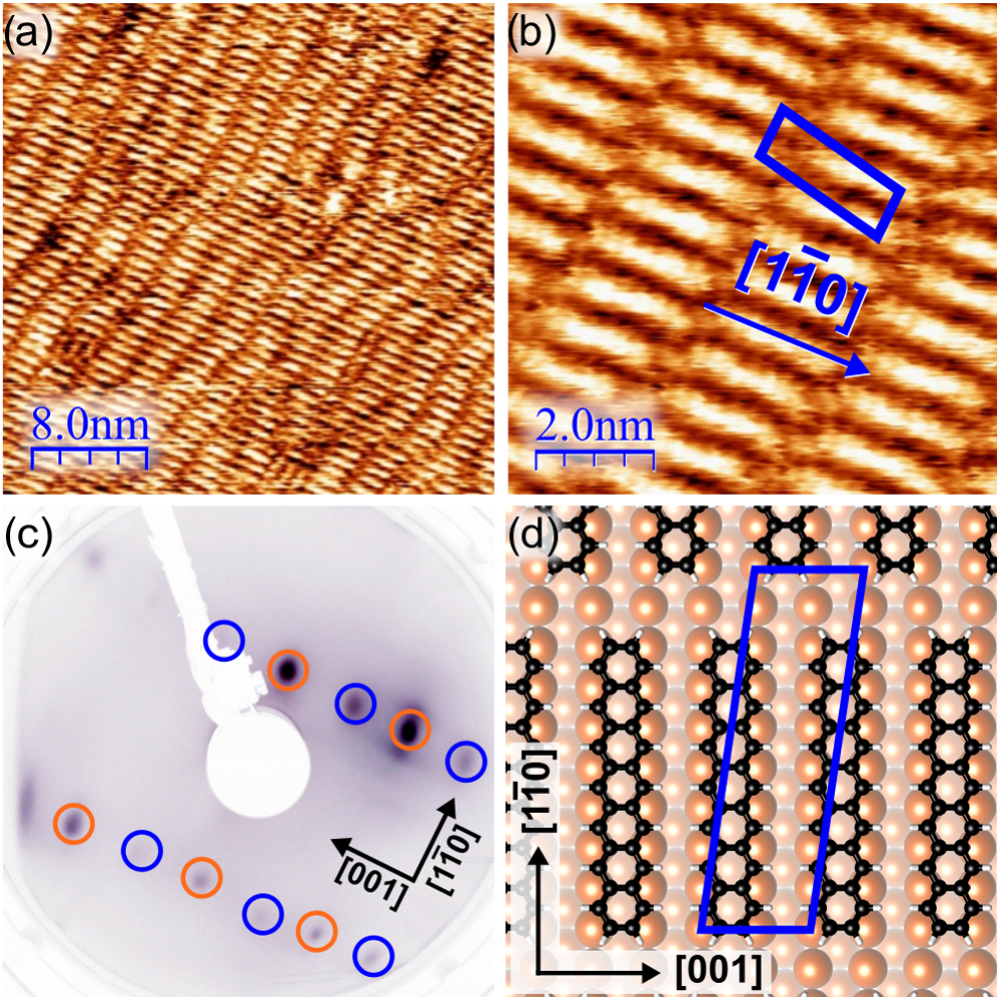
(a) STM image of a monolayer of heptacene on a Cu(110) substrate prepared at room temperature; the STM image was taken with a bias voltage of −0.1 V and a tunneling current of 230 pA. (b) Enlarged STM detail of a well-ordered region after annealing showing heptacene oriented along the favored crystal direction [11̅0] measured with −0.4 V and a tunneling current of 230 pA. (c) LEED pattern of the heptacene/Cu(110) film; orange and blue spots show the Cu(110) surface unit cell and the heptacene film, respectively. (d) Structural model of heptacene on Cu(110) used for the simulations based on STM data.

Employing a thermal cycloreversion of diheptacene isomers as suggested by Einholz et al.,^58^ we grow a monolayer heptacene onto a Cu(110) surface. The (110) surface of Cu is characterized by closed-packed Cu rows along the [11̅0] direction, which have previously been shown to favor the growth of oriented films of the shorter acenes tetracene and pentacene,^33,43,59−61^ where, in all cases, molecules adsorb face-on and tend to either arrange parallel or perpendicular to these rows, denoted as 7A∥row and 7A⊥row, respectively. As shown in the STM images in Figure 1a,1b, we are indeed able to orient the molecules along a preferred adsorption conformation and obtain an ordered monolayer. We find that heptacene, with its long axis along the Cu rows, i.e., along the [11̅0] direction, predominantly arranges in stacks where neighboring stacks are arranged in a staggered manner (cf. Figure 1b). Such an arrangement is also supported by LEED measurements (Figure 1c), where the heptacene pattern (blue) is half the Cu-unit cell (orange) along the [001] direction. This corresponds to molecules occupying every second Cu row. The structural order along the [11̅0] direction is less pronounced and we suggest that the majority of the molecules form no specific long-range periodicity in this direction. STM images also indicate a slight bending of heptacene, which is characteristic of acenes on metal surfaces^61−63^ with their central benzene rings closer to the surface as illustrated in the Supporting Information Figure S4.

Based on the LEED data and STM images, we have constructed a structural model of the adsorption geometry of 7A/Cu(110) depicted in Figure 1d. Using this commensurate monolayer structure with the unit cell shown in blue, we have optimized the interface structure on the van der Waals-corrected DFT/GGA level.^52^ We find the most favorable adsorption configuration of heptacene to be indeed oriented along the [11̅0] row direction with its benzene rings on the hollow adsorption site of the Cu(110) surface (cf. Figure 1d). The calculated adsorption energies for all considered sites and orientations are summarized in Table 1. In agreement with the STM observation, we find the 7A∥row alignment to be more stable by about 0.34 eV than the 7A⊥row configuration and that the hollow site is favored over the bridge adsorption site. It should be noted that the overall adsorption energies include contributions from charge rearrangements, as reflected in the work function changes, as well as from van der Waals interactions that are more sensitive to the local geometric arrangements of carbon atoms relative to substrate atoms.

**Table 1:** Work Functions, Φ, and Adsorption Energies, *E*_ad_, of All 7A/Cu(110) Interfaces Calculated with PBE + D3^a^

	7A∥Cu rows		7A⊥Cu rows	
	*E*_ad_ (eV)	Φ (eV)	*E*_ad_ (eV)	Φ (eV)
bridge	–6.245	3.77	–5.383	3.89
hollow	–6.369	3.40	–6.025	3.81

a Note that the work function of clean Cu(110) is computed to be 4.31 eV.

The work function (WF) change upon forming an organic/metal interface is a key parameter that sheds light on the surface quality and the electronic structure of the interface. Several effects contribute to the work function modification. On the one hand, molecules adsorbed on the surface generally reduce the WF of the clean metal substrate by the Pauli push-back effect. On the other hand, electron transfer from the metal to the molecular layer leads to a surface dipole pointing in the opposite direction and, hence, increases the work function. Moreover, changes in the molecular structure induced by the adsorption may result in an intrinsic dipole of the molecule that also contributes to the overall work function change.^10^

We assess the WF of our systems by performing UPS experiments. For the clean Cu(110) surface, we measured a work function of about 4.43 eV, in good agreement with values from the literature (4.48 eV),^64,65^ assuring the desired cleanliness. With increasing heptacene coverage, the work function decreases until it reaches a value of 3.60 ± 0.10 eV at full monolayer coverage. This experimentally observed change of ΔΦ = (−0.83 ± 0.10) eV compares well to the calculated WF change of ΔΦ = −0.91 eV for the energetically most favored adsorption configuration (hollow site for 7A∥row), i.e., a reduction from 4.31 eV for the bare Cu(110) surface to 3.40 eV for the molecule–metal interface. By analyzing the charge density rearrangements upon adsorption of heptacene on Cu(110) in our theoretical model,^12^ we are able to decompose this overall WF change into several contributions. The bending of the molecule is responsible for a WF drop of ΔΦ_bend_ = −0.39 eV, which can be rationalized from the adsorption geometry depicted in the Supporting Information Figure S4b. The adsorption-induced charge rearrangements, the so-called bond dipole, lead to a ΔΦ_bond_ = −0.52 eV, which accounts for the competing effects of Pauli push-back and charge transfer. By further taking into account a computed net charge transfer of 1.89 electrons as obtained from a Bader charge analysis, and using a simple capacitor model, we estimate Φ_bond_ to arise from the combination of a push-back effect of Φ_push-back_ = −1.43 eV and an opposite dipole of Φ_CT_ = +0.91 eV due to the electron transfer (compare Supporting Information Table S1). It is important to note that the overall simulated work function changes for the other less favorable adsorption configurations are markedly smaller by about 0.4 eV. Moreover, the charge analysis indicates a significantly reduced net charge transfer into heptacene for the perpendicular adsorption orientation.

**Figure 2:**
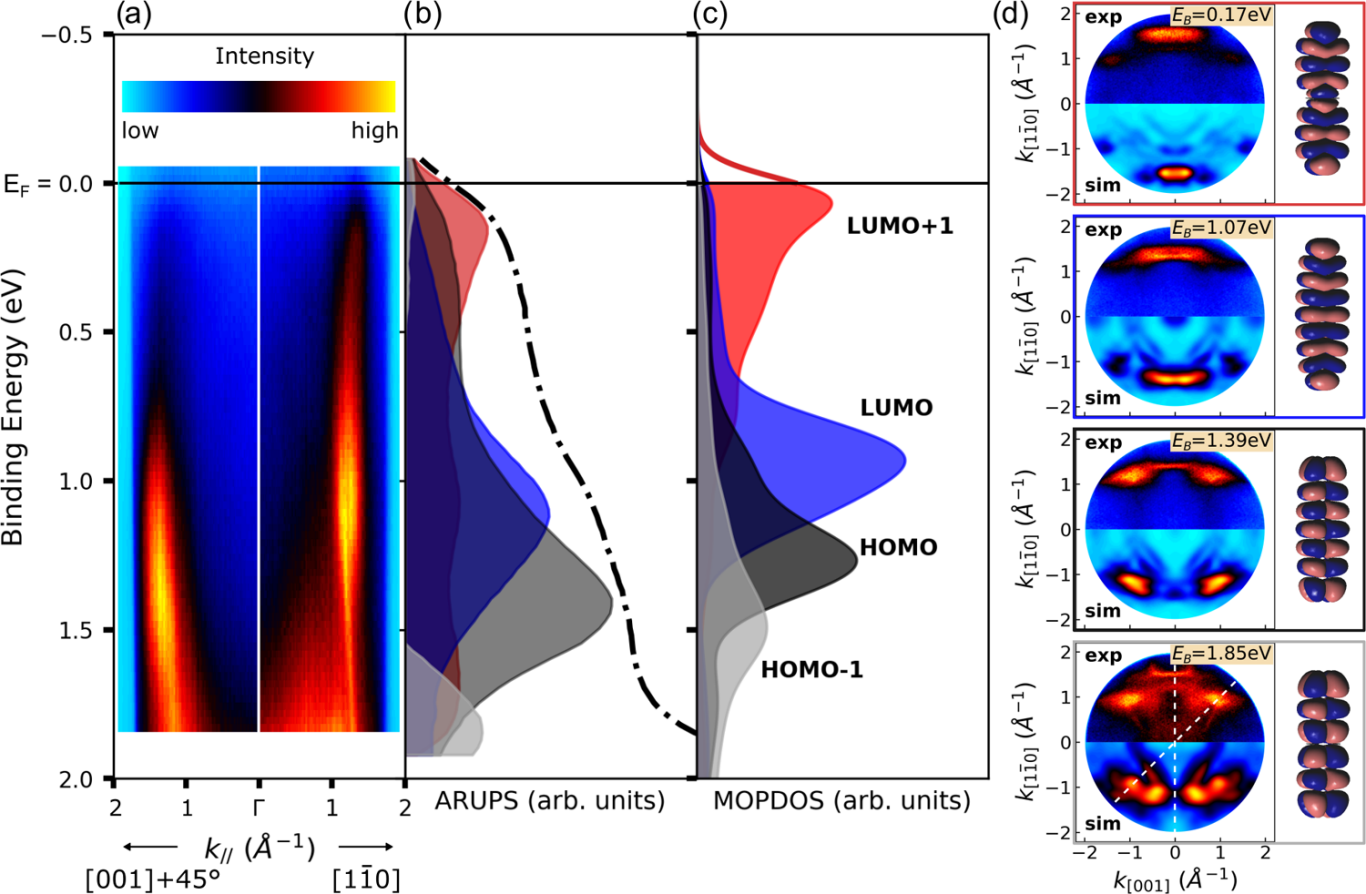
(a) Experimental ARUPS bandmaps along the two directions [11̅0] (from Γ to the right) and [001] +45° (from Γ to the left). (b) Experimental angle-integrated energy distribution curve (dashed line) and the respective orbital contributions obtained by deconvolution of the experimental data cube *I*(*E*_B_, *k*_*x*_, *k*_*y*_) (gray = HOMO – 1, black = HOMO, blue = LUMO, red = LUMO + 1). (c) Calculated molecular orbital projected density of states (MOPDOS) for 7A/Cu(110), multiplied by a Fermi function (*T* = 400 K). (d) Comparison of experimental (top halves) and simulated (bottom halves) momentum maps of 7A/Cu(110) taken at the binding energies corresponding to the maxima of the respective peaks. The corresponding real space electron distributions of the Kohn–Sham molecular orbitals, which we associate with the momentum maps, are shown on the side.

To gain deeper insights into the electronic structure of the heptacene monolayer on Cu(110), we analyze the full angular and energy dependence of our ARUPS data, which are collected in Figure 2. Energy distribution maps, i.e., photoemission intensity maps as a function of the binding energy and the momentum component parallel to the surface, or so-called bandmaps, are depicted in Figure 2a. For an energy window from the Fermi edge to the onset of the Cu-d band at about 2 eV binding energy, we have recorded a complete data cube of bandmaps consisting of *I*(*E*_kin_, *k*_*x*_, *k*_*y*_). The presented bandmaps are cuts through the data cube along two different azimuths, namely, along the Cu row direction [11̅0] (from Γ to right) and for a direction at 45° between the principal substrate azimuths denoted as [001] +45° (depicted from Γ to the left). The chosen azimuths correspond to directions parallel to the long axis of heptacene and 45° to it, respectively, which have been shown to be optimal for detecting emission signatures of the frontier orbitals of acenes.^33,43^ Specifically, the highest occupied molecular orbital (HOMO) is observed along the [001] +45° direction, while the LUMO is visible along [11̅0]. The bandmaps suggest that the HOMO is centered around a binding energy of about 1.4 eV, while the LUMO, being filled upon charge transfer from the metal, has its maximum slightly below 1 eV and extends up to the Fermi energy. This interpretation is supported by our DFT model of the 7A/Cu(110) interface when computing the density of states projected onto the molecular orbitals (MOPDOSs) for the energetically favored configuration (Figure 2c). We indeed find the HOMO (dark gray) and filled LUMO (blue) to be in close vicinity to the binding energies derived from the band maps. Interestingly, the calculation suggests that not only the LUMO but also the LUMO + 1 gets partially filled upon adsorbing heptacene on Cu(110). It is important to note that such a LUMO + 1 occupation is only predicted for the most favorable adsorption configuration, hollow 7A∥row, while the other three adsorption configurations listed in Table 1 only exhibit LUMO occupation (compare Figure S6 in the Supporting Information).

Electron transfer from Cu to oligoacenes has already been observed for pentacene/Cu(110).^43^ However, in the present system with the former LUMO positioned significantly below and new patterns arising at the Fermi level, we observe what could be regarded as a reduction of the molecule, where the Cu surface serves as a coordination partner. Actually, given the higher electron affinity of heptacene, a strongly interacting interface was to be expected. Nevertheless, considering oligoacenes as prototypical electron-rich molecules with electron density above and below the aromatic system, further charge accumulation in the π-system may seem counter-intuitive. To substantiate the unexpected finding of a LUMO + 1 occupation, we measure and simulate constant binding energy momentum maps, that is, maps of the photoemission intensity at a fixed binding energy as a function of *k*_[11̅0]_ and *k*_[001]_. Figure 2d shows momentum maps at four characteristic binding energies, where the upper half of each map depicts the experimental data, while the lower half shows the simulated map computed for the 7A/Cu(110) interface. The characteristic emission features in momentum space and their specific *k*-values are in good agreement with predicted maps and allow us to clearly identify the observed emissions in the bandmaps from bottom to top to the HOMO – 1, HOMO, LUMO, and the LUMO + 1, whose real space orbitals calculated for gas-phase heptacene are also shown in the insets.

In fact, using the Fourier transform of these four electron densities and the entire experimental data cube, that is, photoemission intensity as a function of (*E*_B_, *k*_[11̅0]_, *k*_[001]_), we can deconvolute the emissions into molecular orbital contributions.^66,67^ The resulting deconvolution curves are shown in Figure 2b, using the same color code as that for the MOPDOS plot in panel c, together with a *k*_∥_-integrated energy distribution curve (EDC) shown as a black dashed line. The good agreement between the deconvoluted experimental spectra and the MOPDOS from the DFT calculation gives us further confidence in the assignment of the molecular emissions and also in the exceptionally strong surface-induced charge transfer into the LUMO + 1. Note that in the momentum maps, we can distinguish the LUMO + 1 from the LUMO emission primarily due to the larger *k*_[11̅0]_ value of the LUMO + 1’s main maximum compared to the LUMO. This can already be inferred from the *k*_[11̅0]_ band map shown in panel (a). In summary, the strong reactivity of the Cu(110) surface in conjunction with the high electron affinity of heptacene leads to an (almost) complete filling of the LUMO and to a partial occupation of the LUMO + 1, which is in contrast to the findings for 7A/Ag(110), where only evidence for a partly filled LUMO has been obtained.^45^

Our DFT calculations suggest that a possible occupation of the LUMO + 1 strongly depends on the adsorption configuration of heptacene on Cu(110). To test this hypothesis experimentally, we attempt to grow films with heptacene molecules oriented perpendicular to the Cu row direction. Indeed, by modifying the growth conditions, specifically by cooling the substrate with liquid nitrogen during the evaporation, we are able to produce films in which a substantial fraction of heptacene molecules orient perpendicular to the most favorable [11̅0] row direction. Note that such a minority orientation could already be inferred by closely inspecting the bottom part of the STM image shown in Figure 1a and becomes more apparent in additional STM images recorded at liquid nitrogen temperatures shown in the Supporting Information (see Figure S5 in the Supporting Information). In any case, the molecules adopt a face-on adsorption configuration for both orientations.

**Figure 3:**
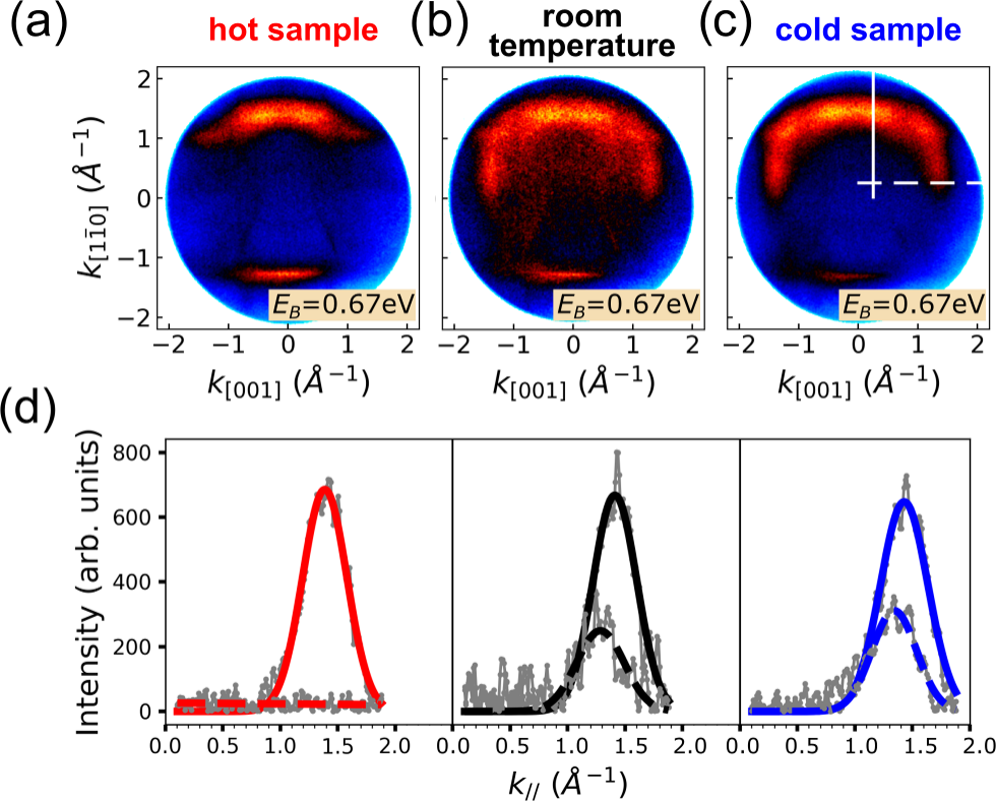
(a–c) ARUPS momentum maps of three samples prepared under different temperature conditions and at the same binding energy (*E*_B_ = 0.67 eV). (d) Change of the intensity of the LUMO emission features with decreasing preparation temperature. The line scans were obtained of background-corrected momentum maps of three differently prepared samples at a binding energy of 0.67 eV along the [11̅0] (solid line) and [001] (dashed line) directions. The experimental data points (gray) are fitted with Gaussian curves and scaled such that the area of the peak along the [11̅0] direction is constant.

The characteristic emission signatures of the LUMO and LUMO + 1 momentum maps with their most prominent emissions along the long molecular axis allow us to disentangle the contributions from the two molecular species, either oriented parallel or perpendicular to the Cu row direction. This is demonstrated in Figure 3, in which we compare ARUPS momentum maps of heptacene films obtained from three different preparation conditions: heated (Figure 3a), room temperature (Figure 3b), and liquid nitrogen (Figure 3c). For the comparison, we have chosen a binding energy of 0.67 eV as, at this energy, the LUMO’s emissions of molecules along both directions are visible. For the heated sample, almost no emissions along [001] are visible, while with decreasing substrate temperature, emissions along the [001] direction can be recognized, which we attribute to heptacene molecules oriented perpendicular to the Cu row direction. When appropriately normalizing linescans along the [11̅0] (white solid line) and [001] (white dashed line) directions, respectively, we obtain the intensity profiles depicted in Figure 3d. The peak connected to the minority [001] alignment of heptacene (dashed lines) clearly increases with decreasing temperature, while the majority alignment with heptacene parallel to [11̅0] still prevails. As a side note, it should be mentioned that on repeating the experiment with another Cu crystal, we find the same qualitative trend with temperature, but the ratio between majority and minority orientations deviates slightly from the data shown here (see Figure S2 in the Supporting Information). This suggests that in addition to the substrate temperature, peculiarities of the used Cu crystal, e.g., the density of step edges and/or kinks, are also important parameters governing the growth of heptacene on Cu(110).

**Figure 4:**
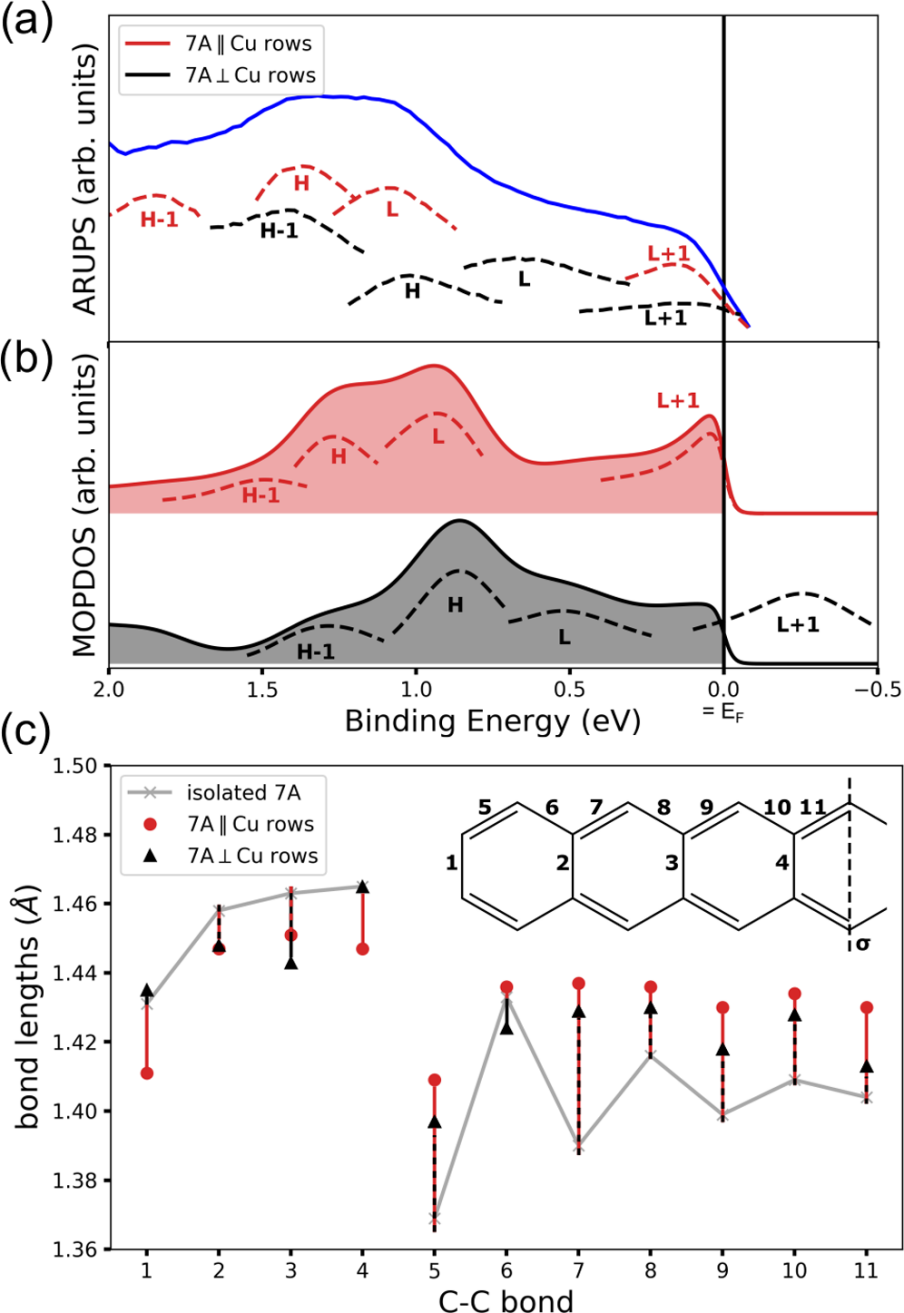
(a) Experimental EDC (solid blue line) for a sample prepared at liquid nitrogen temperatures. The dashed lines show the deconvolution of the experimental ARUPS data orbital contributions (H = HOMO, L = LUMO) of heptacene(7A) oriented along (red) or perpendicular (black) to the Cu(110) rows. (b) Calculated DOS multiplied by the Fermi function (*T* = 100 K) for heptacene adsorbed along (red) and perpendicular (black) to the Cu(110) rows. The dashed lines indicate the individual orbital contributions obtained by MOPDOS analysis. Note that the LUMO + 1 peak of heptacene perpendicular to the Cu(110) rows is shown without the Fermi factor. (c) Calculated bond lengths of an isolated heptacene monolayer (gray) and heptacene along (red dot) and perpendicular (black triangle) to the Cu(110) rows. The bonds are numbered as shown in the inset along with one mirror plane, σ, indicated.

Nevertheless, we can apply photoemission tomography to reveal possible differences in the electronic structure for the two adsorption species of heptacene. To this end, we analyze the photoemission data cube measured for the cold sample, i.e., the intensity as a function of (*E*_B_, *k*_[11̅0]_, *k*_[001]_), over a broader energy range as shown in Figure 4a. While the *k*_∥_-integrated EDC (blue line) shows no apparent differences from the corresponding data of the heated sample (black line in Figure 2b), the deconvolution of the experimental data cube using the theoretical orbitals of heptacene shown in Figure 2d, oriented either along or perpendicular to the Cu rows, is able to reveal the differences in the electronic structure for the two adsorption species. The individual orbital contributions (from HOMO – 1 to LUMO + 1) are plotted as red dashed lines for 7A∥Cu rows and as black lines for the minority 7A⊥Cu species.

As expected, the energy-level positions for the majority 7A∥Cu species are consistent with the deconvolution result for the heated sample discussed earlier (Figure 2b). However, for the 7A⊥Cu species, we observe a significant shift of ∼0.4 eV toward lower binding energies for the orbital contributions of HOMO – 1, HOMO, and LUMO. Moreover, the deconvolution indicates no contribution of the LUMO + 1 for this minority species. These findings are supported by DFT calculations of heptacene on Cu(110) along the two directions.

The results are summarized in Figure 4b, which shows the density of states multiplied by the Fermi function together with the MOPDOS analysis for heptacene along (red) and perpendicular (black) to the Cu(110) rows. The filling of the curves indicates the occupation of the molecular levels. The simulations reproduce the experimental results astonishingly well, with a calculated energy shift of ∼0.4 eV. Moreover, the LUMO + 1 of heptacene along [001] is indeed empty according to the calculations and, fittingly, no emission signatures of this particular orbital have been observed in the experiment. While subtle site dependencies of the electronic structure of heptacene have been observed previously for a Ag substrate,^45^ further analysis of the current 7A/Cu(110) interface suggests that the strong shift toward higher binding energies is facilitated by the almost perfect matching of the surface unit cell of the Cu(110) substrate with the length of one benzene ring. This commensurability ensures that all seven benzene rings of heptacene occupy very similar adsorption sites.

A direct consequence of the charge transfer from the substrate to the molecule can be seen when analyzing the molecular bond length changes upon adsorption of heptacene. Figure 4c compares the calculated bond lengths for a freestanding, neutral monolayer of heptacene (gray), with the ones for heptacene adsorbed on Cu(100) either parallel (red circles) or perpendicular (black triangles) to the Cu rows. The charge transfer into heptacene tends to equalize the bond lengths where the effect is clearly more pronounced for the 7A∥row species with the LUMO + 1 occupation, which is in line with an increased net charge transfer (Table S1 in the Supporting Information). Details of the observed changes can be rationalized by inspecting the nodal structure of the LUMO and LUMO + 1 (see orbital images in Figure 2d). For instance, by the occupation of LUMO and/or LUMO + 1, the additional electron density in formerly electron-poor regions shortens the bond lengths 1–4, while the additional nodes of the LUMO and LUMO + 1 perpendicular to the long molecular axis elongate bonds 5–11.

## Conclusions

A monolayer of heptacene, a member of the long-chain acene family, was successfully prepared on Cu(110) substrates employing a thermal cycloreversion of diheptacene isomers. Angle-resolved ultraviolet photoemission spectroscopy (ARUPS), LEED, and STM measurements prove epitaxial growth and the formation of a highly ordered monolayer film of heptacene on Cu(110). Photoemission tomography reveals the energy-level alignment and identifies an electron transfer from Cu(110) into the formerly unoccupied LUMO and LUMO + 1 orbitals of the organic molecule. The ARUPS momentum maps further indicate the existence of two molecular species on the surface orientated either along or perpendicular to close-packed Cu rows of the (110) surface, the ratio of which can be altered by controlling the film preparation temperature. Despite the fact that both heptacene species adsorb face-on, we observed unexpectedly large differences in their electronic structures. Molecules oriented perpendicular to the rows undergo charge transfer into the LUMO, which was to be expected owing to the large electron affinity of heptacene. However, molecules oriented parallel to the Cu rows exhibit a pronounced shift of the molecular states, leading to an additional occupation of the LUMO + 1. All findings are fully consistent with the densities of states and adsorption geometry calculated by density functional theory, which has proven indispensable to clarify the interplay of various mechanisms taking place upon adsorbing heptacene on Cu(110).

Our results present heptacene molecules in a much different state than usually found in noble gas matrices or current on-surface synthesized arrangements. Moreover, they demonstrate that with the choice of a suitable metal surface and growth conditions, the electronic properties of the molecule can be tuned by a simple face-on rotation without changing the general chemical environment. We further interpret the significant net charge transfer in the present system as stabilization of heptacene and thereby hope to initiate more in-depth studies about the reaction behavior of this formerly unapproachable molecule.

## Supporting Information Available

ARUPS polarization factor; full momentum maps; temperature-dependent LUMO signal; adsorption geometry; surface characterization of a liquid nitrogen-cooled heptacene film; MOPDOS of all adsorption configurations; and work function change (PDF)
