# Supplementary material for: Demonstrating the Impact of the Adsorbate Orientation on the Charge Transfer at Organic–Metal Interfaces
Source: J Phys Chem C Nanomater Interfaces. 2021 Apr 27;125(17):9129–37. doi: 10.1021/acs.jpcc.1c01306 (PMC8154845; doi:10.1021/acs.jpcc.1c01306)
Supplement: Supplementary file 1 — jp1c01306_si_001.pdf [file jp1c01306_si_001.pdf]

Supporting Information for: Demonstrating the Impact of the Adsorbate Orientation on the Charge Transfer at Organic-Metal Interfaces

Thomas Georg Boné,[†,‖] Andreas Windischbacher,[†,‖] Marie S. Sättele,[‡,¶] Katharina Greulich,[‡] Larissa Egger,[†] Thomas Jauk,[§] Florian Lackner,[§] Holger F. Bettinger,[¶] Heiko Peisert,[‡] Thomas Chassé,[‡] Michael G. Ramsey,[†] Martin Sterrer,[†] Georg Koller,[†] and Peter Puschnig[*,†]

†*Institute of Physics, University of Graz, 8010 Graz, Austria*

‡ *Institute of Physical and Theoretical Chemistry, University of Tübingen, 72076 Tübingen, Germany*

¶ *Institute of Organic Chemistry, University of Tübingen, 72076 Tübingen, Germany*

§*Institute of Experimental Physics, Graz University of Technology, 8010 Graz, Austria*

‖*these authors contributed equally to the work*

E-mail: peter.puschnig@uni-graz.at

Supporting Information

ARUPS polarization factor

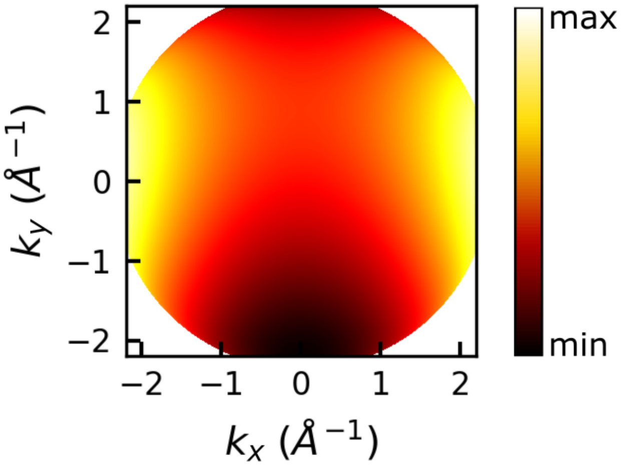

Figure S1: Polarization dependent factor $|\vec{A}\cdot\vec{k}|^2$ of the ARUPS momentum maps as a superposition of 69.4% s-polarized and 30.6% p-polarized light. Emission features are enhanced in brighter regions and suppressed in darker regions.

Within the framework of photoemission tomography and the plane wave final state approximation, momentum maps can be understood as the product of the molecular orbital's Fourier transform, and a weakly angle-dependent polarization factor, $|\vec{A}\cdot\vec{k}|^2$. The latter depends on the direction and polarization of the incident light described by $\vec{A}$ and the electron's emission direction described by its wave vector $\vec{k}$. In our experiments, the He discharge light is directed onto the surface at an angle of 22° between light and surface parallel level. For the measurements in this work, the light is cast on the surfaces along the y axis in positive direction. It consists of s-polarized (69.4%) and p-polarized (30.6%) components leading to a polarization factor shown in Figure S1.[1] As a result of this $|\vec{A}\cdot\vec{k}|^2$ term, features parallel to the [001] direction (horizontal direction in Figure S1) are slightly enhanced. It should that the $|\vec{A}\cdot\vec{k}|^2$ reasonably well accounts for the directivity of emissions from molecules. Thus molecular emissions yield higher signal intensity on the upper half of momentum maps. Due to the limitations of the plane wave final state approximation for describing emissions

from metallic states, however, metal emissions are typically higher on the lower half of the momentum maps.

Full momentum maps

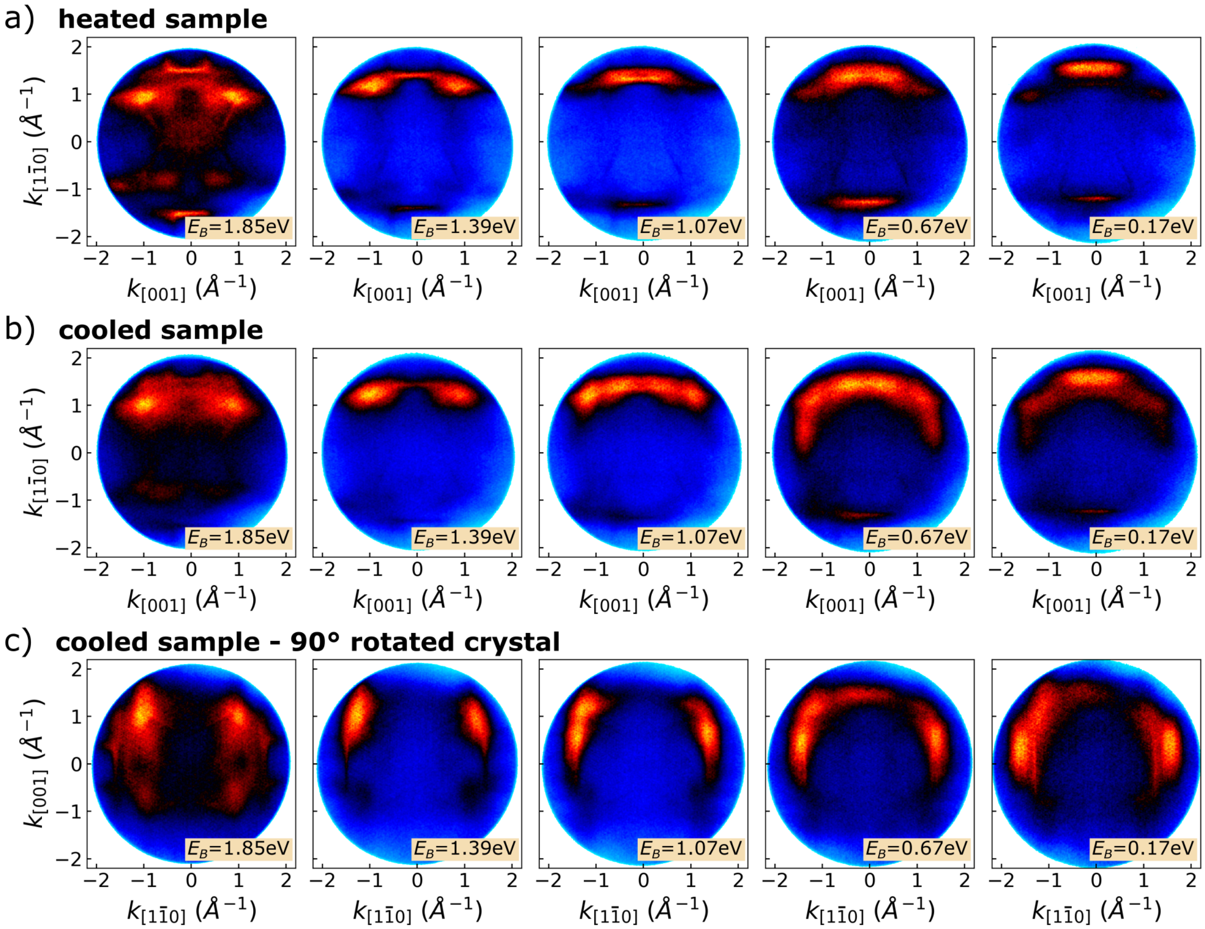

Figure S2: Momentum maps at the same binding energies of three differently prepared samples. 1 ML heptacene on Cu(110) prepared a) at elevated temperature, b) at liquid nitrogen temperature, c) at liquid nitrogen on a 90° rotated crystal

Figure S2 summarizes momentum maps of the three differently prepared samples of 7A/Cu(110), on which the main manuscript focuses on. The binding energies are chosen at the positions of prominent emission features according to Figure 4. Figure S2a displays the maps of heptacene on a Cu surface, which was heated during molecule deposition and the maps are equivalent to Figure 2d and Figure 3a. Figure S2b shows the momentum

maps of a heptacene film on a Cu-substrate, which was cooled with liquid nitrogen during the deposition. The visual broadening of the emission patterns compared to Figure S2a originates from additional emissions of molecules oriented along a second orientation, i.e.: along [001]. To exclude wrong findings from spurious intensities due to the polarization effect of the probing light, we prepared a heptacene film under the same conditions on a 90° rotated substrate. Its momentum maps are shown in Figure S2c and their agreement with Figure S2b supports our original conclusion.

Growth temperature dependence of the molecular orientation

Changing the growth temperature can change the molecular orientation on the Cu(110) substrate. At elevated temperature, the molecules are oriented along the [1-10], whereas at lower temperatures the amount of molecules oriented in [001] increases. To demonstrate this effect, we have further evaluated the temperature dependence of the emission signals of heptacene on the 90° rotated Cu(110) surface. Figure S3 shows the momentum maps of two samples prepared under different temperature conditions: (a) at room temperature and (b) cooled with liquid nitrogen. We have not measured a heated sample as we only observe a single molecular orientation along $[1\bar{1}0]$ at hot temperatures. The maps are taken at a binding energy of 0.67 eV to investigate the relation between the LUMO's emission of molecules along $[1\bar{1}0]$ and [001]. For better comparison, the line-scans are normalized in accordance with Figure 3d of the main text. In particular for the cooled sample, we see a significant change to Figure 3d with both adsorption orientations almost in a 1:1 relation. Considering the data collected for Figure 3d and Figure S3 were measured on two different sample crystals, we suggest that irregularities of the surface influence the growth of domains along [001].

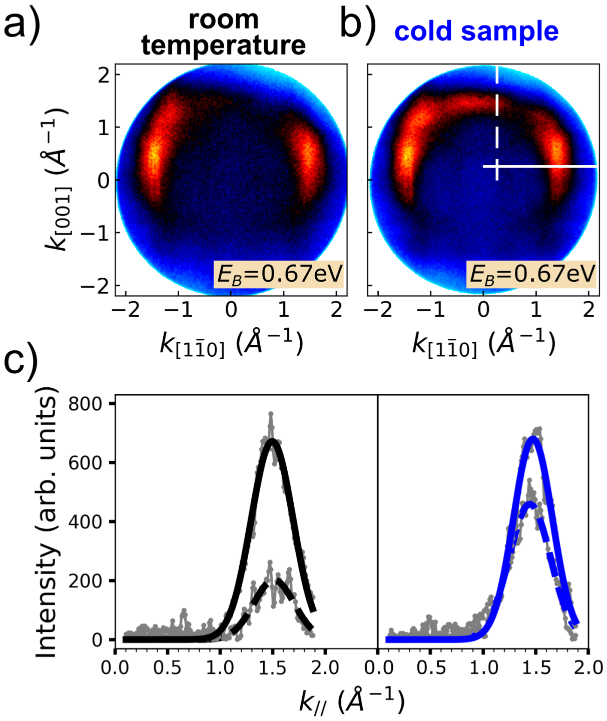

Figure S3: a) and b) ARUPS momentum maps of two samples prepared under different temperature conditions on a Cu-crystal, which is rotated by 90° compared to Figure 3 taken at the same binding energy (E_B=0.67eV). c) Change of the intensity of the LUMO emission features with decreasing preparation temperature. The line scans were taken of background corrected momentum maps of the two differently prepared samples at a binding energy of 0.67 eV along the $[1\bar{1}0]$ (solid line) and [001] (dashed line) direction. The experimental data points (gray) are fitted with Gaussian curves and scaled such, that the area of the peak along the $[1\bar{1}0]$ direction is constant and in agreement with Figure 3.

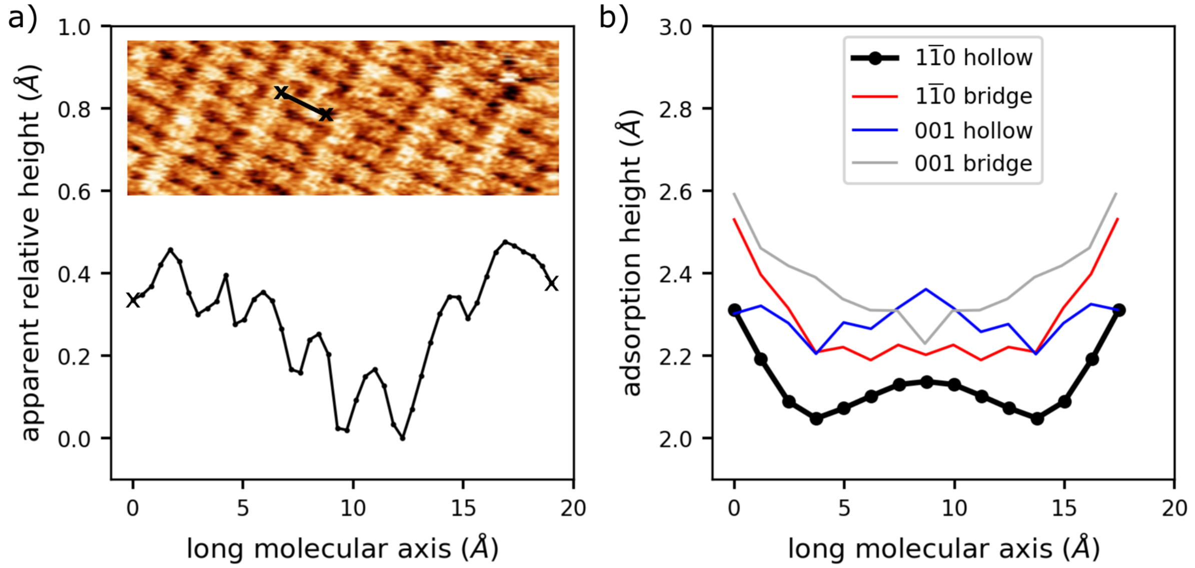

Figure S4: a) High-resolution STM image of an heptacene monolayer on Cu(110) taken at a bias voltage of -0.1 V and a tunneling current of 700 pA in comparison to the dedicated z-profile. b) side view of the carbon backbone of four calculated adsorption configurations of heptacene/Cu(110).

Adsorption geometry

The strong interaction between heptacene and Cu leads to geometric distortions of the molecule. Figure S4 shows a high-resolution STM image of the heptacene monolayer together with the apparent height profile over the length of one molecule. This suggests a bend of heptacene of ∼0.4 Å. A bend of this magnitude is also seen in the DFT calculations in Figure S4, where the carbon backbones for four adsorption configurations are shown. It should also be emphasized, that the most favoured calculated adsorption site, "[1$\bar{1}$0] hollow", which exhibits the largest charge transfer, is significantly closer to the surface by ∼0.2 Å than all other configurations.

Surface characterization of liquid Nitrogen cooled heptacene film

We have investigated the heptacene film prepared under liquid nitrogen temperatures with STM and LEED. Compared to Figure 1, the STM image in Figure S5a shows an increasing amount of molecules oriented along the [001] direction. However, we were not able to obtain

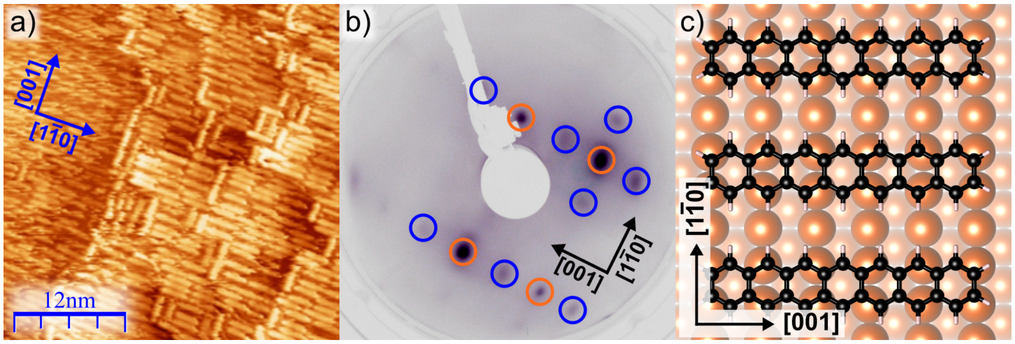

Figure S5: a) STM image of a monolayer heptacene prepared on a Cu(110) substrate cooled with liquid nitrogen taken at a bias voltage of -0.1 V and a tunneling current of 730 pA. b) LEED pattern of a monolayer heptacene prepared on a Cu(110) substrate cooled with liquid nitrogen; orange spots mark the Cu(110) unit cell, blue spots originate from the molecular arrangement. c) structural model of heptacene along the [001] direction.

local images of larger domains. The LEED pattern in Figure S5b provides more information on the averaged molecular arrangement, where orange and blue spots mark the Cu(110)-surface unit cell and the heptacene film, respectively. At cold temperatures, additional points appear along the [1$\bar{1}$0] direction at a third of the Cu(110) cell in reciprocal space. A molecule every third Cu-unit cell corresponds to the smallest real space distance, at which rows of heptacene oriented along [001] can arrange (compare Figure S5c), suggesting that such areas form in not negligible size.

MOPDOS of different adsorption sites and overlayer structures

In the main part of our manuscript, the computations of the 7A/Cu(110) interface are based on experimentally determined overlayer structures. Here, we present supplementary results for the molecular orbital projected DOS (MOPDOS) for four additional overlayer structures with the aim to mimic a range of coverages of 7A on Cu(110) (compare Table S1). In total, we have thus analyzed the MOPDOS for five overlayer structures and four different adsorption configurations characterized by the adsorption sites, bridge or hollow, and the molecular orientations, [1$\bar{1}$0] or [001], respectively. The results are summarized in Figure S6.

Here, the full MOPDOS curves for the HOMO-1, HOMO, LUMO and LUMO+1, re-

Table S1: Epitaxial matrices $(m_{11}, m_{12}/m_{21}, m_{22})$ for all simulated overalyer structures and their corresponding molecular coverages.

	7A‖Cu rows		7A⊥Cu rows	
Configuration	Matrix	coverage (molecule/nm^2)	Matrix	coverage (molecule/nm^2)
exp	(2,0/1,9)	0.623	(6,1/0,3)	0.623
I	(2,3/0,9)	0.623	(6,0/2,3)	0.623
II	(2,0/0,9)	0.623	(6,0/0,3)	0.623
III	(3,0/0,10)	0.374	(7,0/0,4)	0.401
IIII	(4,0/0,11)	0.255	(8,0/0,5)	0.281

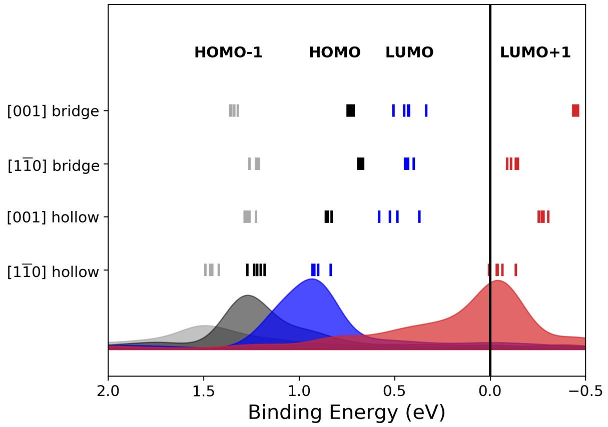

Figure S6: Density of states projected onto the molecular orbitals (MOPDOS) for four different adsorption configurations sampling five different overlayer structures as listed in Table S1. The binding energies corresponding to the maxima of the respective orbital contributions from HOMO-1 to LUMO+1 are marked. The full MOPDOS of the experimental overlayer structure along the [1$\bar{1}$0] direction with the molecule in the hollow-site is shown at the bottom. The color code of the molecular orbitals is in agreement with Figure 2 of the main manuscript.

spectively, are only shown for the experimental overlayer structure and the $[1\bar{1}0]$-hollow configuration, that is, for the energetically most favorable configuration (filled areas). In the addition, the peak positions of the HOMO-1, HOMO, LUMO and LUMO+1 curves are indicated by a bar. For all other overlayer structures and adsorption configurations, only the corresponding peak positions are indicated in the figure by respective bars. Two main conclusions can be drawn from this analysis. First, it demonstrates the unique character of interaction on the "hollow" adsorption site along the $[1\bar{1}0]$ direction. Only for this configuration, the LUMO+1 receives significant charge and, moreover, LUMO, HOMO and HOMO-1 are shifted to larger binding energies compared to the other three adsorption configurations. Second, the fact that there is only a minor spread in the MOPDOS peaks (bars) for different overlayer structures shows that the energy level alignment at the 7A/Cu(110) interface is primarily determined by the adsorption configuration (site and orientation) and that the molecular packing only plays a secondary role.

To verify the applicability of our projection scheme, we compare the calculated partial charge densities for our heptacene/Cu-interfaces in the energy range of the MOPDOS peaks of Figure 4 in the main manuscript to the charge densities of the freestanding molecular monolayers (Figure S7). With the characteristic lobes of the HOMO, LUMO and LUMO+1 visually distinguishable and recognizeable, we find our MOPDOS analysis well suited for the frontier orbitals.

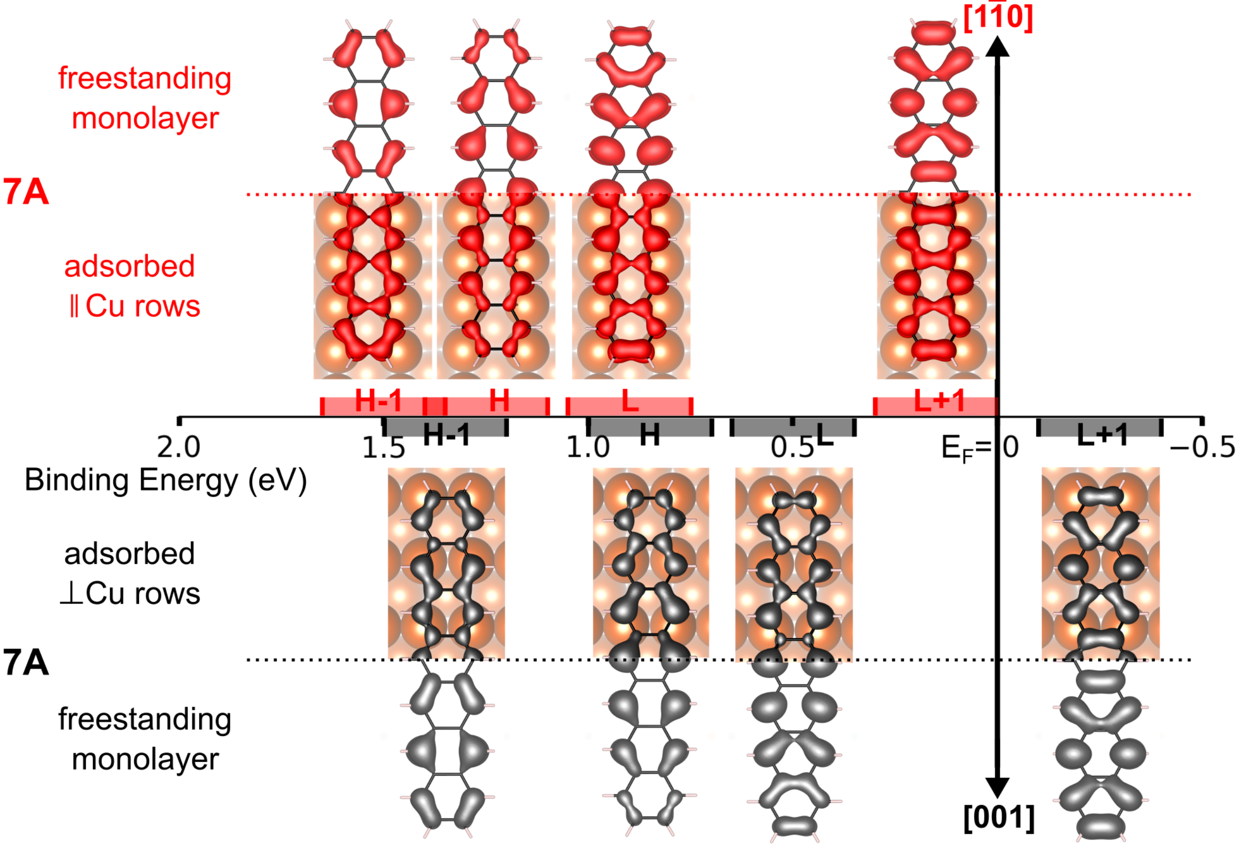

Figure S7: Partial charge densities of the interfaces integrated over the energy range of 0.3 eV around the MOPDOS peaks shown in Figure 4 of the main manuscript, compared to the charge densities of the freestanding molecular monolayer.

Analysis of work function changes

The work function changes upon adsorption of 7A can be partitioned into several contributions according to the following equation

$$\Delta\Phi_{\text{tot}} = \Delta\Phi_{\text{bend}} + \underbrace{\Delta\Phi_{\text{CT}} + \Delta\Phi_{\text{push-back}}}_{=\Delta\Phi_{\text{bond}}}. \tag{1}$$

Here, $\Delta\Phi_{\text{bend}}$ denotes the work function change due to a molecular dipole induced by geometrical changes of the molecule upon adsorption, $\Delta\Phi_{\text{CT}}$ represents the increase of the work function due to charge transfer from the surface to the molecule, and $\Delta\Phi_{\text{push-back}}$ reduces the work function as electrons are pushed back towards the surface by Pauli repulsion. The latter two effects can be summarized as the charge rearrangements due to electronic interactions between molecules and substrate and are combined in $\Delta\Phi_{\text{bond}}$.

The work function change due to charge rearrangements can be obtained from an analysis of the plane-averaged charge density difference between the molecule-substrate interface and its organic and inorganic subsystems. Figure S8 depicts the total plane-averaged charge density (gray line) as well as the charge densities, red (blue) denoting electron accumulation (depletion), for four adsorption configurations. By solving the one-dimensional Poisson equation, the corresponding change in the electro-static potential can be obtained (green dashed line) leading to the potential step $\Delta\Phi_{\text{bond}}$. The results are summarized in Table S2.

It is interesting to note that despite significantly different work functions of the four investigated adsorption configurations (compare Table 1 of main manuscript), at first sight, there is only little difference in their plane-averaged density differences (Figure S8). To substantiate this unexpected finding, we further analyze the different contributions to the work function change introduced in Eq. 1. It is clear that the separation between the influence of charge transfer and push-back is somewhat ambiguous, in particular for strongly interacting systems with chemisorption character like the present one. Nevertheless , we want to elaborate on the range of the total work function changes presented in Table S2 as

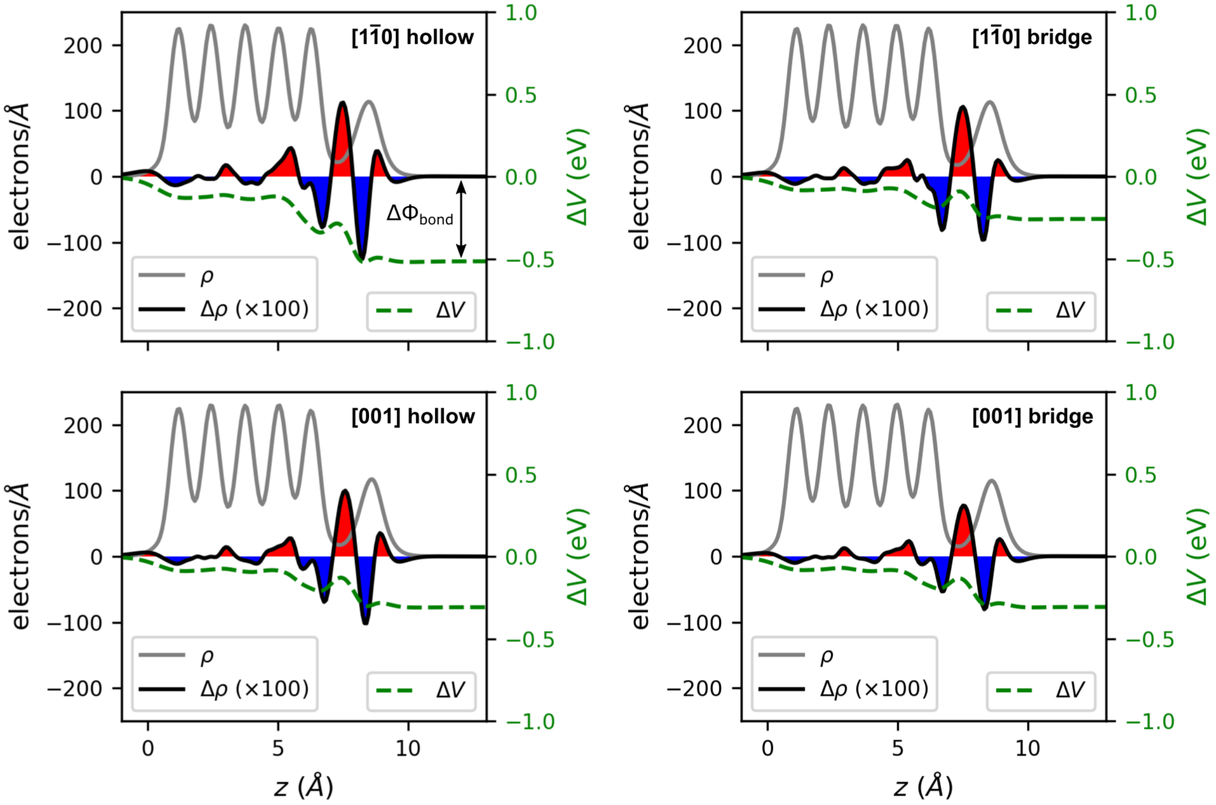

Figure S8: Total charge densities, ρ, of all four investigated adsorption configurations, charge density differences, $\Delta\rho$, of the molecule-substrate interfaces and their subsystems and changes in the electrostatic potentials, ΔV. Red (blue) denotes gain (loss) in electron density upon adsorption.

Table S2: Calculated work function (WF) changes, $\Delta\Phi_{\mathrm{tot}}$, upon adsorbing heptacene on Cu(110) for four adsorption configurations as obtained from PBE+D3 calculations. Decomposition into WF change, $\Delta\Phi_{\mathrm{bend}}$, due to adsorption induced bending of the molecule, WF change due to charge separation, $\Delta\Phi_{\mathrm{CT}}$, WF change due to push-back effect, $\Delta\Phi_{\mathrm{push\text{-}back}}$. The averaged adsorption height, d, as well as the net charge transfer (CT) from a Bader charge analysis in units of the electron charge are also given.

	7A‖Cu rows		7A⊥Cu rows	
	Hollow	Bridge	Hollow	Bridge
$\Delta\Phi_{\mathrm{bend}}$ (eV)	-0.39	-0.28	-0.19	-0.11
$\Delta\Phi_{\mathrm{CT}}$ (eV)	0.91	0.75	0.71	0.64
$\Delta\Phi_{\mathrm{push\text{-}back}}$ (eV)	-1.43	-1.01	-1.02	-0.95
$\Delta\Phi_{\mathrm{tot}}$ (eV)	-0.91	-0.54	-0.50	-0.42
d (Å)	2.14	2.29	2.29	2.39
CT (e)	1.89	1.45	1.37	1.18

one initially might suspect configurations with smaller charge transfer to have higher work function changes.

The intrinsic dipole moment of heptacene in the direction perpendicular to the surface, $\Delta\Phi_{\mathrm{bend}}$, was calculated as the vacuum potential step for a freestanding monolayer of the molecule already in its distorted geometry. To futher disentangle $\Delta\Phi_{\mathrm{bond}}$ into its individual contributions, we performed a Bader charge analysis[2–5] on the interface systems yielding an approximation for the excess charge in the adsorpted molecule (cf. last line in Table S2). We employed a simple capacitor model assuming a homogenous negative charge density within the molecular monolayer and a positive counterpart in the metal surface. The influence on the work function calculates according to Eq.(2)

$$\Delta\Phi_{\mathrm{CT}} = \frac{CT \cdot d}{\epsilon_0 \cdot \epsilon_{\mathrm{r}}} \tag{2}$$

where $\Delta\Phi_{\mathrm{CT}}$ is in eV and CT is in C/m^2. The average distance between the molecule and the surface is taken for d, ϵ_0 is the vacuum permittivity and ϵ_{r} represents the estimated dielectric constant of heptacene.[6] We assume the remaining contribution to the total work function change to be $\Delta\Phi_{\text{push-back}}$. Again, it has to be stressed that absolute numbers have to be taken with care considering the approximations in GGA/DFT and post-processing. Note that the actual ϵ_{r} of a heptacene monolayer will most likely be smaller than the bulk value and, thus, our approximated contributions of $\Delta\Phi_{\mathrm{CT}}$ represent lower limits. However, the values in Table S2 follow an expected trend as adsorbates closer to the surface experience stronger charge transfer and at the same time are able to repel more electron density back to the surface. In comparison for the system Xe/Cu(110), a work function change of 0.6 eV was measured and, due to the noble gas character of Xe, solely attributed to the push-back effect.[7] There, the adsorption height is 1 Å larger than for 7A/Cu(110).[8]

References

(1) Brandstetter, D.; Yang, X.; Lüftner, D.; Tautz, F. S.; Puschnig, P. kMap.py: A Python program for simulation and data analysis in photoemission tomography. *Comp. Phys. Commun.* **2021**, *263*, 107905.

(2) Henkelman, G.; Arnaldsson, A.; Jónsson, H. A fast and robust algorithm for Bader decomposition of charge density. *Computational Materials Science* **2006**, *36*, 354–360.

(3) Sanville, E.; Kenny, S. D.; Smith, R.; Henkelman, G. Improved grid-based algorithm for Bader charge allocation. *Journal of computational chemistry* **2007**, *28*, 899–908.

(4) Tang, W.; Sanville, E.; Henkelman, G. A grid-based Bader analysis algorithm without lattice bias. *Journal of Physics: Condensed Matter* **2009**, *21*, 084204.

(5) Yu, M.; Trinkle, D. R. Accurate and efficient algorithm for Bader charge integration. *The Journal of chemical physics* **2011**, *134*, 064111.

(6) Ejuh, G.; Nya, F. T.; Kamsi, R. Y.; Ndjaka, J. Investigation of the electronic, optoelectronics, and linear and nonlinear optical properties of the molecules heptacene ([7] acene)(C30H18) and [7] acene doped with potassium atom (C30H9K9). *Polymer Bulletin* **2018**, *75*, 637–652.

(7) Crispin, X.; Geskin, V.; Crispin, A.; Cornil, J.; Lazzaroni, R.; Salaneck, W. R.; Bredas, J.-L. Characterization of the interface dipole at organic/metal interfaces. *Journal of the American Chemical Society* **2002**, *124*, 8131–8141.

(8) Caragiu, M.; Seyller, T.; Diehl, R. Adsorption geometry of Cu (110)-(12× 2)-14Xe. *Surface science* **2003**, *539*, 165–170.
